# The Relationship of Redox With Hallmarks of Cancer: The Importance of Homeostasis and Context

**DOI:** 10.3389/fonc.2022.862743

**Published:** 2022-04-22

**Authors:** Faliang Xing, Qiangsheng Hu, Yi Qin, Jin Xu, Bo Zhang, Xianjun Yu, Wei Wang

**Affiliations:** ^1^ Department of Pancreatic Surgery, Fudan University Shanghai Cancer Center, Shanghai, China; ^2^ Department of Oncology, Shanghai Medical College, Fudan University, Shanghai, China; ^3^ Shanghai Pancreatic Cancer Institute, Shanghai, China; ^4^ Pancreatic Cancer Institute, Fudan University, Shanghai, China; ^5^ Department of Thoracic Surgery, Shanghai Pulmonary Hospital, Tongji University School of Medicine, Shanghai, China

**Keywords:** redox homeostasis, antioxidant response, hallmarks of cancer, ROS, Nrf2

## Abstract

Redox homeostasis is a lifelong pursuit of cancer cells. Depending on the context, reactive oxygen species (ROS) exert paradoxical effects on cancers; an appropriate concentration stimulates tumorigenesis and supports the progression of cancer cells, while an excessive concentration leads to cell death. The upregulated antioxidant system in cancer cells limits ROS to a tumor-promoting level. In cancers, redox regulation interacts with tumor initiation, proliferation, metastasis, programmed cell death, autophagy, metabolic reprogramming, the tumor microenvironment, therapies, and therapeutic resistance to facilitate cancer development. This review discusses redox control and the major hallmarks of cancer.

## 1 Introduction

The normal physiological activities of an organism are carried out in a state of equilibrium, which is regulated by a precise and complex system. Redox reactions are also part of this balance. The redox system is divided into an oxidation and a reduction system. In brief, as mitochondrial respiration produces reactive oxygen species (ROS), the reduction system of the cells produces antioxidant enzymes to remove ROS to restore a physiological level so that redox is always in a dynamic balance, called “redox homeostasis”. However, when affected by various factors, the redox system is dysregulated, leading to an imbalance between oxidation and reduction. Excessive ROS are produced and cannot be cleared in a timely manner. Increased production of different ROS leads to molecular damage, a condition called “oxidative stress” (1). The increased levels of ROS have deleterious effects on cellular homeostasis, structures, and functions. Thus, disruption of the cellular redox homeostasis promotes various pathological processes, including cancer (2).

Redox homeostasis is not only a requirement for physiological processes in normal cells but also a pursuit of cancer cells. In normal cells, the antioxidant system plays an important role in eliminating excess ROS; it does so also in cancer cells, and the antioxidant effect is even amplified in these cells. The occurrence and development of tumors are inseparable from the effects of redox conditions. During tumorigenesis, ROS generation and elimination are involved throughout the processes of tumorigenesis and tumor development. Cancer cells are characterized by high concentrations of ROS. However, why can cancer cells still undergo uncontrolled proliferation and invasion? The tight regulation of redox in cancer cells contributes to their survival. Studies have demonstrated that cancer cells amplify the activity of antioxidant systems to scavenge ROS to combat oxidative stress (3). In addition, reduced stress is involved in chemo- and radioresistance of cancer cells. Therefore, understanding the role of redox regulation in the initiation, development, and treatment of tumors can provide strategies and ideas for treating tumors and overcoming drug resistance. In this review, we address the oxidation and reduction systems, the relationship of these systems with hallmarks of cancer, and the involvement of the redox system in the treatment of cancer.

## 2 The Oxidation System and Oxidative Stress

ROS is the general term for labile, reactive, and partially reduced oxygen derivatives, which include hydrogen peroxide (H_2_O_2_), superoxide anion ( $O_{2^{-}}$ ), hydroxyl radical (•OH), singlet oxygen (^1^O_2_), and hypochlorous acid (HOCl) (4). ROS can be generated by multiple endogenous and exogenous factors (5). Mitochondria are the major source of endogenous ROS, based on the role of the respiratory chain in the internal mitochondrial membrane during oxidative phosphorylation (OXPHOS), an event that generates adenosine triphosphate (ATP) and reduces molecular oxygen (O_2_) to water *via* the electron transfer chain (6). The electron transfer chain consists of four enzyme complexes: nicotinamide adenine dinucleotide (NADH):ubiquinone (Q) oxidoreductase (complex I), succinate–Q reductase (complex II), ubiquinol–cytochrome c reductase (complex III), and cytochrome c oxidase (complex IV) (7). Complex I and III, where the electron potentials relevant to oxygen reduction undergo dramatic changes, are the two prime sites for ROS production. Electron leakage from complexes I and III results in the production of $O_{2^{-}}$ (8). Furthermore, mutation or deficiency of subunits of succinate dehydrogenase (SDH, also known as complex II) can result in increased mitochondrial $O_{2^{-}}$ generation and more severe oxidative stress, and can lead to cellular genomic instability and transformation into cancer cell phenotypes (9). Studies have shown that SDHB-deficient cell lines exhibit increased mitochondrial activity and lipid peroxidation levels, as well as elevated mitochondrial copper and cytoplasmic iron levels, and more ROS in the cytoplasm and mitochondria (10). Other contributors to superoxide radical (i.e., $O_{2^{-}}$ ) production in mitochondria include glycerol 3-phosphate dehydrogenase, pyruvate dehydrogenase (PDH), and 2-oxoglutarate dehydrogenase (11). Manganese superoxide dismutase (MnSOD) converts $O_{2^{-}}$ to H_2_O_2_ in the mitochondrial matrix. H_2_O_2_ in the mitochondrial matrix can be further turned to •OH by mitochondrial aconitase *via* the Fenton reaction (12).

**Figure 1:**
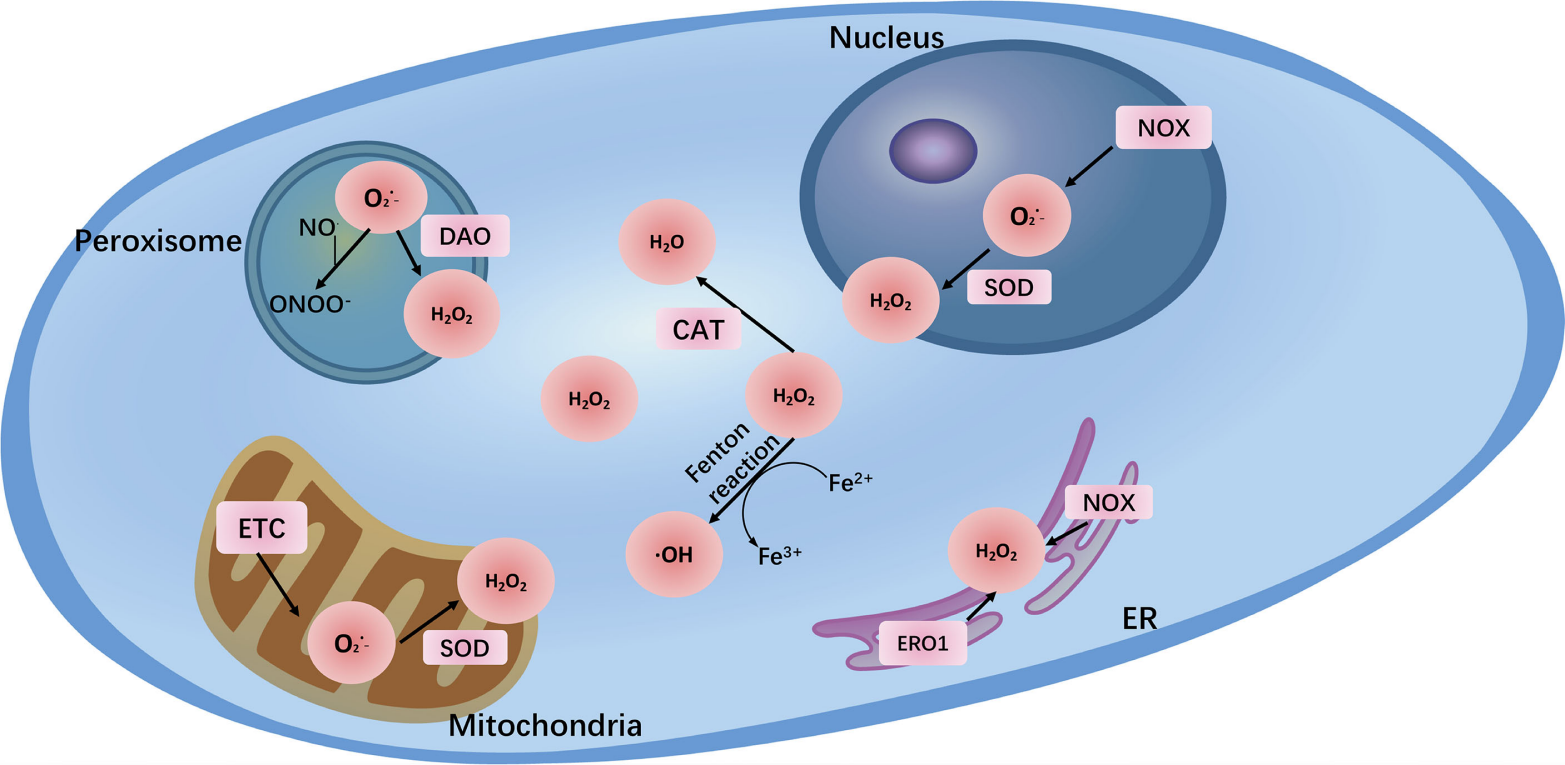
The major reactive oxygen species ( $O_{2^{-}}$ , H_2_O_2_, ONOO^-^) and their sites of production within cells. The main endogenous sources of ROS are mitochondrial electron transport chain (ETC) and NADPH oxidases (NOXs). In addition, peroxisome and endoplasmic reticulum (ER) also generate ROS. Transition metal ion iron nonenzymatically catalyzes ROS generation *via* the Fenton reaction.

In addition, many enzymes are also related to the production of ROS, mainly those of the nicotinamide adenine dinucleotide phosphate (NADPH) oxidase (NOX) family, which contains 7 members: NOX1, NOX2, NOX3, NOX4, NOX5, dual oxidase 1 (DUOX1), and DUOX2 (13). NOXs are membrane-linked enzymes that reduce oxygen to $O_{2^{-}}$ by transferring one electron from NADPH to FAD across biological membranes (14). Other enzymes include diamine oxidase (DAO) (15), cyclooxygenases (COXs), and lipoxygenases (LOX) (16). Additionally, peroxisome and endoplasmic reticulum (ER) stress can lead to the generation of a wide range of ROS under physiological or pathological conditions (17). Notably, transition metal ion iron also nonenzymatically catalyzes ROS generation *via* the Fenton reaction, in which process Fe^2+^ reacts with H_2_O_2_ to generate •OH, which can damage biomolecules such as DNA and proteins. Therefore, iron is recognized as a major source of ROS and is growingly viewed as an important inducer and mediator of cell destruction in various pathological conditions through the production of ROS. Iron-induced oxidative stress has been regarded as one of the risk factors for various cancers (18) (**Figure 1**).

**Figure 2:**
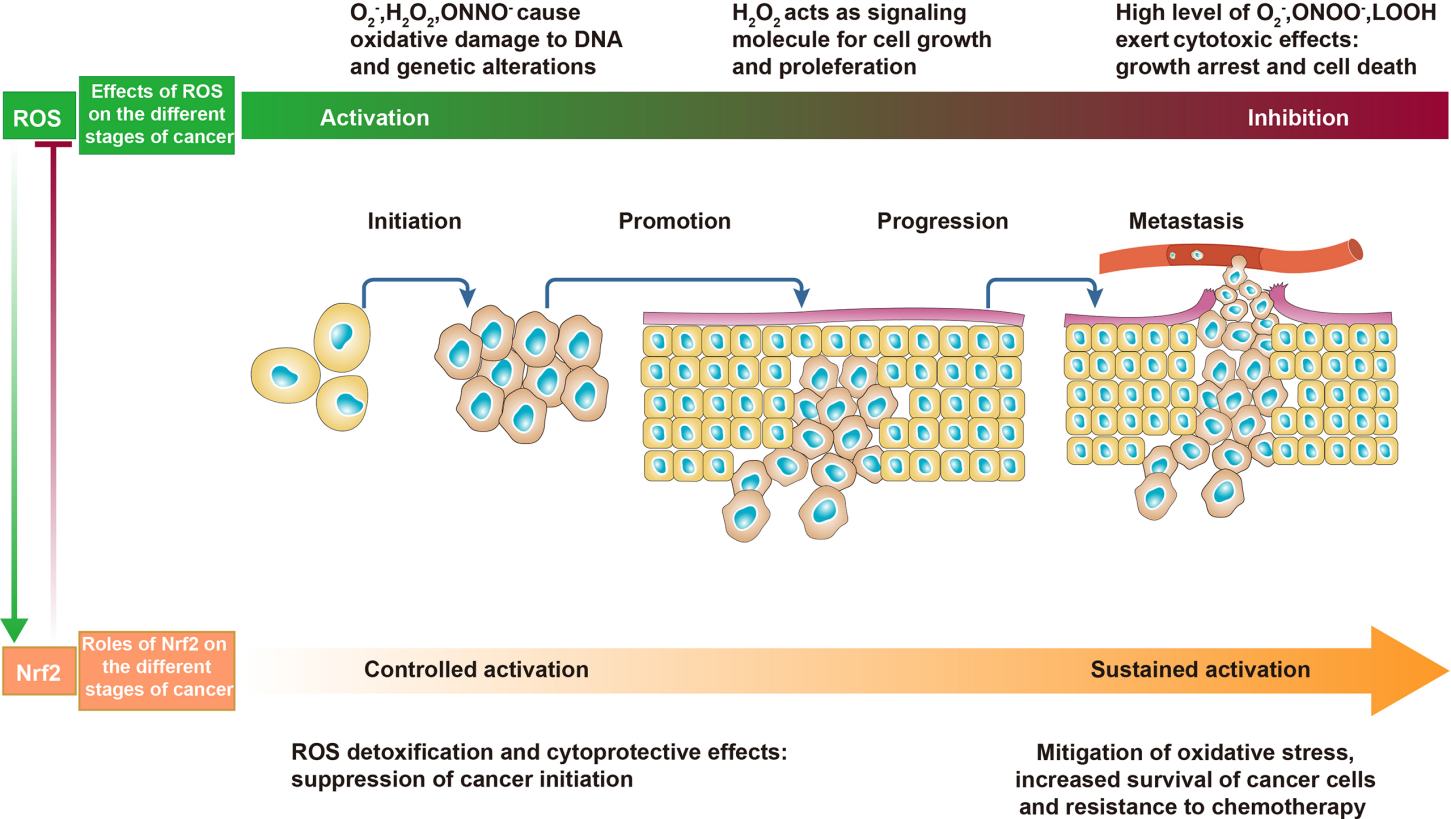
The effects of reactive oxygen species (ROS) and Nrf2 on cancer. In the early stages of cancer, appropriate levels of ROS act as initiators and mediators of carcinogenesis and cancer promotion. In the advanced stages of cancer, excessive ROS promote cancer cell death and are detrimental to cancer progression. In normal cells and precancerous cells, controlled activation of Nrf2 decreases ROS and inhibits cancer initiation. In malignant and metastatic cells, sustained activation of Nrf2 counteracts oxidative stress to promote cancer progression.

During oxidative stress, the abovementioned various enzymes and mechanisms are dysregulated, resulting in excessive ROS accumulation. In both normal cells and cancer cells, ROS exhibit a concentration-dependent duality. At physiological levels, ROS act as second messengers in cell signaling and are essential for various biological processes in normal cells. When ROS are elevated, they may damage biological macromolecules such as DNA, proteins, and polyunsaturated fatty acids (PUFAs), change the entire cell structure and function, and even cause genetic mutations that induce susceptibility to malignancy. In cancer cells, ROS also act as a “double-edged sword”, at low to moderate levels, inducing genetic changes, and are crucial for cancer initiation, proliferation, and progression, as well as the development of therapeutic resistance (19). In other words, appropriate concentrations of ROS are indispensable for cancer cell redox homeostasis and mediating the initiation of cellular processes including proliferation, growth, differentiation, and migration. On the contrary, high concentrations of ROS have cytotoxic effects, such as activating apoptosis and inhibiting resistance to anticancer therapy (5). Regarding the dual feature of ROS, innovative approaches to decrease or increase ROS levels have potential for cancer prevention or treatment (**Figure 2**).

## 3 The Reduction System and Its Activation

The reduction system, also called the antioxidant system, which keeps ROS at a low level to ensure redox homeostasis in normal cells, plays an important role in protecting the integrity of cellular structure and function. The antioxidant system converts ROS to less reactive molecules to counteract the harmful effects of ROS. The term antioxidant refers to any substance that slows down, limits, or represses the production of ROS, detoxifies those already generated by devoting their own electrons, and thereby suppresses the damaging reactions of toxic oxidants (20). Accordingly, antioxidants are classified into endogenous and exogenous enzymatic and nonenzymatic antioxidants in accordance with their pattern of function, their site of action, and their source. The endogenous enzymatic antioxidant system comprises superoxide dismutase (SOD), glutathione peroxidase (GPX), glutathione S-transferase (GST), catalase (CAT), heme oxygenase (HO), peroxiredoxin (Prx, PRDX), thioredoxin (Trx), paraoxonase (PON), etc. The nonenzymatic antioxidant system includes glutathione (GSH), NADPH, NADH, vitamin C, vitamin E, and melatonin, among others.

Reductive stress is defined as the upregulation of the cellular antioxidant system to produce and accumulate excess reducing substances. An increase in the GSH/GSSG, NADH/NAD+, NADPH/NADP+, or cysteine/oxidized cysteine (disulfide) ratio or upregulation of antioxidase can neutralize ROS, driving cells away from oxidative stress and toward reductive stress (21). In both normal cells and cancer cells, reductive stress actually plays a protective role, but the outcomes after protection are different. In normal cells, a surplus of ROS damages cell structure and function, and the antioxidant system protects cells from damage and inhibits tumor initiation by alleviating oxidative stress and acting cytoprotective functions. In cancer cells, to prevent cell destruction and death caused by excessive intracellular ROS, cancer cells abnormally activate and increase the transcription of antioxidant enzymes to inhibit oxidative stress and prevent cell cycle arrest and apoptosis caused by excessive ROS (22).

The transcription factor nuclear erythroid 2-related factor (Nrf2) is a core redox-sensitive regulator that behaves as a crucial redox switch (23). Under normal physiological situations, Nrf2 is degraded to inhibit its expression and activity by Kelch-like ECH-associated protein 1 (KEAP1), a substrate adaptor for the Cullin-3 (Cul-3)-dependent E3 ubiquitin ligase complex. However, under oxidative conditions, a distinct set of cysteine residues in KEAP1 are modified by ROS and electrophiles, which obstructs its conjunction with Nrf2 and the Cul-3 ubiquitin ligase. As a consequence, Nrf2 separates from KEAP1 and transfers from the cytoplasm to the nucleus, where Nrf2 associates with the small protein Maf to form the complexes that bind to and activate antioxidant response elements (AREs) in target genes (24). AREs regulate the expression of downstream detoxification enzymes and cytoprotective proteins, such as thioredoxin reductase 1, NAD(P)H quinone dehydrogenase 1, HO-1, SOD, GPX, CAT, and phase II detoxification enzymes (25). Nrf2 has opposing roles in cancer based on context (**Figure 2**). During cancer initiation, Nrf2 can prevent carcinogenesis by increasing the expression of antioxidants to scavenge ROS. However, as cancer progresses, under conditions of excessive ROS accumulation in cancer cells, Nrf2 is a critical regulator of the antioxidant system to protect cancer cells from damage by ROS (26). Nrf2 is hyperactivated and its expression is upregulated in cancer cells for their survival, promoting disease progression. Abnormal overexpression of Nrf2 has been observed in numerous different types of cancer, such as pancreatic cancer, lung cancer, breast cancer, ovarian epithelial carcinoma, and endometrial cancer (27–30). Other pathways can also exert redox effects through Nrf2. For instance, the phosphatidylinositol 3-kinase (PI3K)/protein kinase B (Akt) axis, which is important for cell growth, proliferation, and survival, can regulate Nrf2-mediated ROS detoxification. Akt-mediated phosphorylation on a specific serine residue activates the Nrf2 signaling pathway, which then facilitates cancer cell growth and survival by protecting cells from damage by excessive levels of ROS (31).

In addition, redox proteins peroxiredoxins and thioredoxins are important antioxidant systems in cancer cells that scavenge ROS and maintain redox homeostasis. Studies have shown that peroxiredoxins and thioredoxins are overexpressed in a variety of cancers and are involved in multiple stages of cancer development (32, 33). Knockdown of PRDX6 results in mitochondrial dysfunction, increased ROS levels, and cell cycle arrest in HepG2 hepatocarcinoma cells (34). The NADPH/NADP+ ratio is also a valuable antioxidant defense mechanism; NADPH functions as an electron donor to increase the reductive potential of GSH and thioredoxins. The NADP+/NADPH ratio regulates glucose-6-phosphate dehydrogenase (G6PD) and 6-phosphogluconate dehydrogenase (6PG) activity to increase NADPH production for oxidative stress resistance (35). Therefore, cancer cells can defend themselves against excessive ROS by antioxidant regulation through various mechanisms, and this ability is important for their proliferation and progression.

## 4 Redox Regulation and the Hallmarks of Cancer

### 4.1 Oxidative Stress Affects the Initiation of Cancer

**Figure 3:**
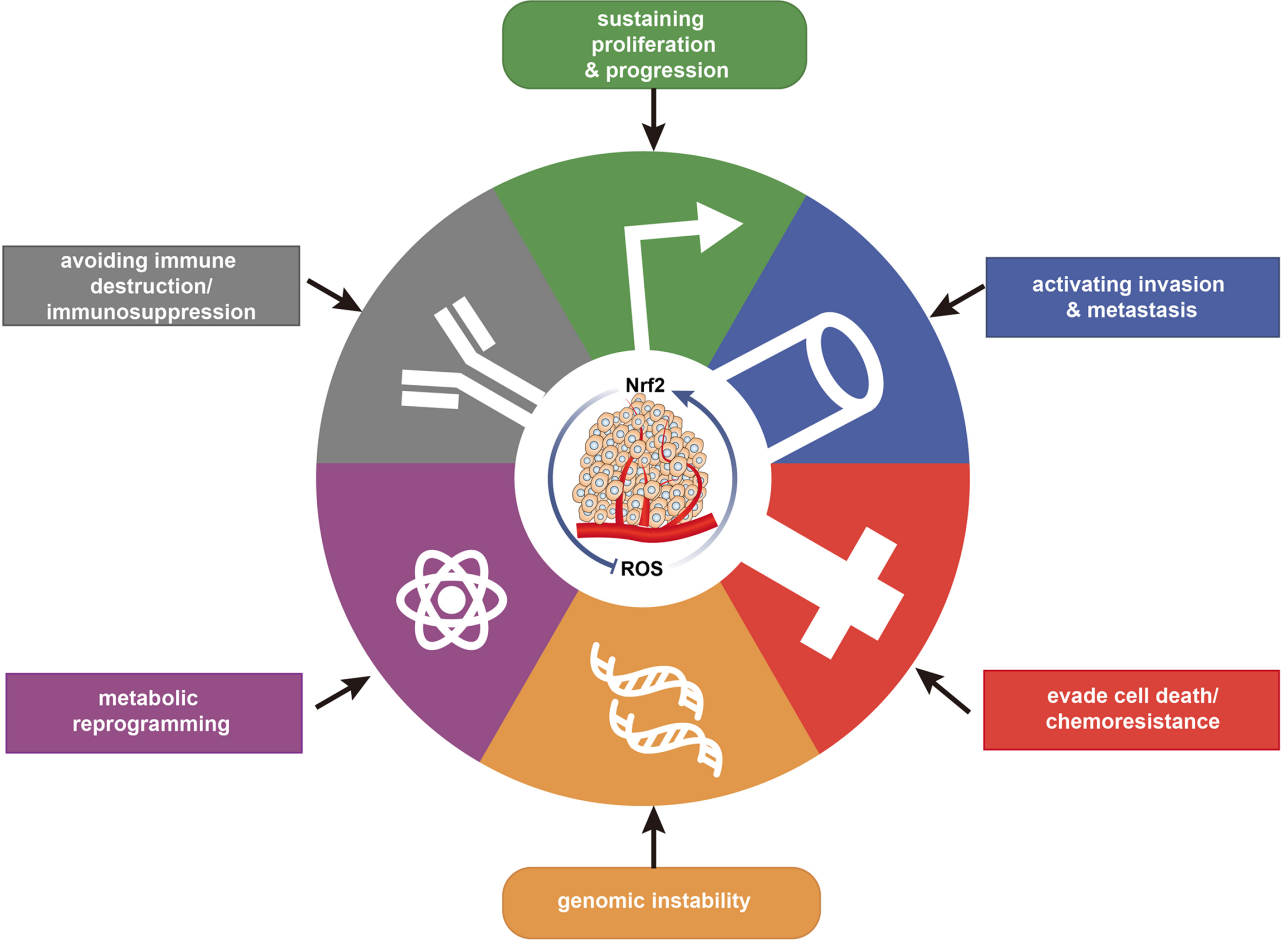
Relationship of redox with the major hallmarks of cancer. Redox regulation is associated with the hallmarks of cancer, maintaining redox homeostasis and impacting cancer progression.

The physiological activities of normal cells are carried out in a state of redox homeostasis (**Figure 3**). Various factors cause redox imbalance, leading to oxidative stress. Excessive ROS in normal cells is related to tumorigenesis. Tumorigenesis is the formation of a new tumor from malignant transformed somatic cells, one of the first stages of which is DNA damage. Excessive ROS can directly induce oxidative DNA damage and cause genomic instabilities, leading to mutations. This effect is seen especially in proto-oncogenes and tumor suppressor genes, in which unrepaired mutations can trigger tumor initiation (36). Types of DNA damage include DNA double-strand breaks and the formation of oxidative DNA adducts such as mutagenic 8-oxo-7,8-dihydro-2′-deoxyguanosine (8-oxodG) and 8-hydroxy-2′-deoxyguanosine (8-OHdG). These compounds are widely considered markers of endogenous DNA oxidative damage as well as risk factors for cancer initiation (37). 8-OHdG causes a shift in histone methylation from an activated to a more inhibited status, which in turn results in aberrant methylation of tumor suppressor genes, leading to their inactivation and dysfunction in human hepatocarcinogenesis (38). In response to oxidative stress, Nrf2 prevents carcinogenesis through the rapid enzymatic modification and efflux of chemical carcinogens and the elimination of ROS or alleviation of oxidative damage through expression of its target genes (39). However, the intense damaging effects of oxidative stress in normal cells can cause redox imbalances. Therefore, disruption of redox homeostasis contributes to the initiation and development of cancer (40).

### 4.2 Sustaining Cancer Proliferation and Progression *via* the Redox Status

Cancer cells strive for a balanced redox environment to support their growth, proliferation, and progression. Disruption of redox homeostasis is detrimental to cancer cells. From the perspective of oxidation, the effect of ROS on tumors depends on the context—namely, the concentration, species, and site of action—of ROS. For instance, low to moderate concentrations of ROS have positive effects on cancer cells because they can act as signaling molecules involved in cancer cell growth, proliferation, and metabolism. As one of the lines of evidence, downregulation of SDHC expression resulted in decreased mitochondrial SDH activity and increased ROS levels in hepatocellular carcinoma (HCC) cells, thereby promoting HCC cell growth and metastasis *in vitro* and *in vivo* (41). ROS can induce tumor cell proliferation and enhance survival *via* numerous pathways. ROS oxidize and inactivate mitogen-activated protein kinase (MAPK) phosphatases, activating the MAPK pathway and promoting cancer cell growth, proliferation, and development (42). In addition, the ROS-activated MAPK pathway regulates the synthesis and activation of activator protein 1 (AP-1). AP-1 is a transcription factor that influences cell proliferation, invasion, cell cycle, and apoptosis and is activated by c-Jun N-terminal kinase (JNK), extracellular regulated protein kinases 1/2 (ERK1/2), and p38 (43). Activation of AP-1 is beneficial to cell proliferation owing to its enhancement of the expression of growth-stimulatory genes, such as cyclin D1, and simultaneous inhibition of the cell cycle inhibitor p21 (44). In addition to activating transcription factors, ROS regulate the expression of genes related to cell proliferation and progression *via* epigenetic modifications. Activation of protein kinase C (PKC) by phorbol ester 12-O-tetradecanoylphorbol-13-acetate (TPA) can augment NOX2 activity following ROS production in MCF7 breast cancer cells. These ROS can induce histone H3 acetylation in the slug promoter region and thus upregulate slug expression. Slug is a main transcription factor involved in epithelial–mesenchymal transition (EMT), and its expression favors cell survival, proliferation, migration, and invasion (45). However, ROS perform these functions when present at the appropriate level and in the early stage of tumor formation. In other words, ROS in cancer cells can exert their cancer-promoting effects only under redox homeostasis. When the level of ROS is too high, ROS can lead to death and cell cycle arrest in cancer cells. Cunning tumor cells also have strategies to circumvent these effects and balance the excess ROS.

ROS levels in cancer cells are significantly higher than in normal counterparts partly due to mitochondrial dysfunction and hypermetabolism. Excessive ROS are destructive to cancer cells. To survive under conditions of excessive oxidative stress, cancer cells are constantly trying to enhance their antioxidant defenses to eliminate excess ROS. Malignant or metastatic cells utilize reductive stress to promote their viability. Hyperactivation of Nrf2 is a common mechanism. Sustained accumulation or activation of Nrf2 and subsequent upregulation of antioxidants results in an environment conducive to the survival and growth of malignant cells (46). KEAP1-mutant non-small-cell lung cancer (NSCLC) induces Nrf2 to activate the expression of thioredoxins and peroxiredoxins, which are critical for Nrf2-dependent growth and proliferation in KEAP1-mutant cell lines (47). By maintaining the redox homeostasis, Nrf2 restrains ROS-mediated activation of apoptosis-inducing JNK/p38 signaling cascades, thereby contributing to cancer cell proliferation and growth (48). In addition, cancer cells activate other signaling pathways and transcription factors to promote their proliferation and growth under conditions of redox homeostasis. FOXO proteins belong to the forkhead transcription factors subfamily, which participates in diverse cellular events and plays an important role in regulating cellular processes such as cell proliferation and apoptosis. To date, four FOXO members have been discovered in mammals: FOXO1, FOXO3a, FOXO4, and FOXO6. ROS regulate the activity and function of FOXO proteins, which are involved in the regulation of intracellular redox state. In response to excessive ROS, acetylated FOXO3a translocates from cytoplasm to nucleus, where it is deacetylated through interaction with nuclear sirtuin 1 (SIRT1) (49). An inadequate supply of reducing equivalent, such as NADH, can lead to the activation of SIRT1, a regulator of cellular redox activity (50). When deacetylated FOXO proteins become abundant in the nucleus, the expression of genes implicated in cell cycle and DNA repair is upregulated, as well as the expression of antioxidant enzymes, including SOD and CAT (51). FOXOs are essential for SIRT1-dependent cancer cell vitality. In addition, the overexpression of peroxisome proliferator-activated receptor δ (PPARδ) has been found to facilitate colorectal cancer cell growth and promotion by increasing the expression of vascular endothelial growth factor (52). Nuclear factor kappa-light-chain-enhancer of activated B cells (NF-κB) is an important nuclear transcription factor that participates in cellular inflammatory and immune response and regulates DNA transcription, cytokine production, and cell differentiation and apoptosis. Activation of NF-ĸB can be accelerated in a redox-dependent manner *via* pyruvate dehydrogenase kinase 1 (PDK1). Through that mechanism, epithelial growth factor receptor (EGFR) exerts proproliferative effects, which was found to drive the appearance of pancreatic precancerous lesions in Kras mouse models (53). Therefore, maintaining the redox balance is very important for tumor cells. On the one hand, the reduction system maintains ROS at a certain concentration and inhibits the apoptotic effect of ROS on tumors; on the other hand, the level of ROS after redox regulation can allow ROS to exert a protumor effect.

### 4.3 Cancer Cells Resist Oxidative Stress During Metastasis

Metastasis is actually an inefficient and difficult process for tumor cells. Metastatic cancer cells migrate through a variety of environments that are significantly different from that of their tumor origin, such as blood and lymph nodes, resulting in disruption of redox homeostasis through various mechanisms, a partial reason that the vast majority of tumor cells die during metastasis (54). To adapt to oxidative stress, the few surviving metastatic cells undergo reversible changes, thereby acquiring resistance to oxidative stress.

Cancer cells deactivate some metabolic pathways during metastasis, partially to preserve reducing equivalents to control oxidative stress. Some anabolic pathways consume reducing equivalents, weaken the antioxidant system, and aggravate oxidative stress. In turn, oxidative stress can also inhibit cell anabolism by consuming reducing equivalents. For example, reducing equivalents from NADPH are required for lipogenesis; decreasing acetyl-CoA carboxylase limits NADPH consumption by fatty acid synthesis and conserves more NADPH, promoting resistance to oxidative stress and other biological processes (55). Therefore, metastatic neoplastic cells downregulate or block these anabolic pathways to preserve more antioxidants for oxidative stress resistance to facilitate metastasis. Cells with metastatic potential and metastatic cells undergo epigenetic and transcriptional changes, upregulate antioxidant production and enhance the antioxidant capacity. The expression of Prx5 is upregulated in oral squamous cell carcinoma (OSCC) cells, thereby scavenging ROS and promoting OSCC cell invasion and growth (56). Melanoma cells in hypoxic areas of primary tumors demonstrate increased expression of the lactate transporter monocarboxylate transporter 1 (MCT1), which mitigates oxidative stress and improves the vitality of cancer cells in the blood (57). MCT1 upregulates lactate uptake, thereby decreasing the NAD+/NADH ratio, which increases pentose phosphate pathway (PPP) activity, a primary source of NADPH for alleviating oxidative stress (58). Oxidative stress is further increased when metastasizing cancer cells enter the bloodstream (59) because of the high levels of oxidants and fluid shear stress in the blood. Fluid shear stress can induce apoptosis in metastatic breast cancer cells by increasing mitochondrial generation of $O_{2^{-}}$ . Circulating metastatic cancer cells demonstrate upregulated mitochondrial MnSOD expression, converting $O_{2^{-}}$ to relatively stable H_2_O_2_, endowing cells with apoptosis resistance (60). In addition, some metastatic cancer cells enter the blood in clusters, which may promote their survival in the blood partially by decreasing their exposure to oxidants, thus attenuating the damaging effects of ROS (61). To improve the efficiency of metastasis, cancer cells may have an increased tendency to undergo lymphatic metastasis because the lymphatic system facilitates cancer cell migration and invasion. Vascular endothelial growth factor C (VEGFC) and multiple chemokines induce and accelerate metastatic cancer cells entry into lymphatic vessels, promoting metastatic spread. Overexpression of VEGFC in mouse lungs elevates the lymphatic vessel density and contributes to the spread of cancer cells from the lung to other regions (62). Moreover, evidence indicates that melanoma cells in the lymphatic circulation undergo less oxidative stress and form more metastatic lesions than metastatic cells in the blood circulation (63). Lymph contains fewer oxidants than blood and protects metastatic melanoma cells from damage induced by oxidative stress.

Although excessive ROS generally inhibits the survival of cancer cells during metastasis and invasion, ROS also promote tumor metastasis in some contexts. For instance, inhibition of TP53 induced glycolysis regulatory phosphatase (TIGAR), an enzyme that facilitates the influx of glucose into the PPP, and increases the levels of ROS in pancreatic ductal adenocarcinoma (PDAC) cells, resulting in enhanced invasive and metastatic ability of cancer cells (64). Moreover, H_2_O_2_ induces activation of AP-1 in a JNK-dependent manner, leading to upregulation of matrix metallopeptidase 7 (MMP-7) and increased metastasis in human colon cancer cells (65). These effects may depend on the concentration, type, and source of ROS. Moderate concentrations of ROS induce the metastasis of cancer cells. Different kinds of ROS have different roles on cancer cells. For instance, H_2_O_2_ generated *via* mitochondria may contribute to cancer cell metastasis (66), while lipid peroxides generated by membrane lipid peroxidation may inhibit cancer cell migration and invasion (63).

### 4.4 Cancer Cells Evade Death *via* Redox Regulation

Cell death is an intricate process and is considered the endpoint of cell fate for both normal cells and cancer cells. Through extensive and thorough research, cell death modalities have been divided into two categories: accidental cell death and regulated cell death (RCD). The latter type of cell death, predominantly including apoptosis, necroptosis, pyroptosis, and ferroptosis, is characterized by controlled signaling pathways and definite effector mechanisms (67). Various stimuli or signals, including but not limited to oxidative stress, induce regulated death in cancer cells, which is also a mechanism and strategy for tumor treatment. However, cancer cells can avoid death through various methods, collectively called apoptosis resistance, which is a general hallmark of cancer (68). This review mainly describes the control of apoptosis, necroptosis, pyroptosis, and ferroptosis by cancer cells through redox regulation.

#### 4.4.1 Apoptosis Is Influenced by the Redox Status

Apoptosis is a type of caspase-dependent RCD. There are two major patterns of apoptosis, which are mediated by extrinsic (cell death receptors) pathway and intrinsic (mitochondria) pathway (69). The extrinsic pathway is initiated after a death ligand such as FASLG or tumor necrosis factor (TNF) binds to a death receptor such as FAS or tumor necrosis factor receptor 1 (TNFR1), respectively, subsequently inducing the recruitment of the adaptor protein FAS-associated protein with death domain (FADD) and caspase-8 to form death-inducing signaling complexes (DISCs). After its recruitment, caspase-8 becomes activated and triggers apoptosis *via* cleavage of downstream caspases, including caspase-3, caspase-6, and caspase-7. ROS can initiate transmembrane death receptors, such as FAS, tumor necrosis factor-related apoptosis inducing ligand (TRAIL-R1/2), and TNFR1, to trigger apoptosis. The intrinsic pathway can also be activated by high levels of mitochondrial ROS (70), which increase mitochondrial outer membrane permeabilization (MOMP), resulting in the subsequent release of mitochondrial proteins such as cytochrome c into the cytoplasm. Cytochrome c binds to and promotes the oligomerization of apoptotic peptidase activating factor 1 (APAF1) into the apoptosome complex, which recruits and activates caspase-9 (71). Subsequently, caspase­9 cleaves and activates executioner procaspases, which in turn cleave a range of essential substrates to initiate apoptosis. Increased concentrations of ROS can induce apoptosis in pancreatic cancer cells by activating the p38 signal cascade (72). An underlying mechanism for this occurrence is activation of apoptosis signal-regulating kinase 1 (ASK1), which is combined with the reduced form of Trx. After ROS-dependent oxidation of Trx, ASK1 detaches from Trx and is activated, which in turn induces the phosphorylation of p38 and enhances the intrinsic apoptotic pathways (73). In summary, oxidative stress promotes both the intrinsic and extrinsic apoptotic pathways in cancer cells (74). To evade apoptosis, cancer cells upregulate Nrf2 to prevent ROS-mediated activation of apoptosis. Furthermore, Nrf2 directly suppresses apoptosis by increasing the antiapoptotic proteins BCL-2 and BCL-xL, reducing cytochrome c release from mitochondria, and decreasing caspase-3/7 activation (75, 76).

#### 4.4.2 Necroptosis and Pyroptosis

In situations where the catalytic activity of caspases is pathogenically inhibited and apoptosis is blocked, necroptosis serves as a “backup” mode of RCD (77). Necroptosis can be induced by the same stimuli as apoptosis, such as activation of death receptors, but is also mediated by receptor-interacting serine/threonine kinase 1 (RIPK1) (78). Intracellular adaptor elements such as FADD bind to RIPK1 and subsequently RIPK3, resulting in the recruitment and phosphorylation of mixed lineage kinase domain-like pseudokinase (MLKL) to form the necrosome. RIPK3-induced phosphorylation of MLKL leads to its oligomerization and future location at the cytomembrane, promoting membrane destabilization (79). TNFα was found to induce the accumulation of both mitochondrial and NOX1-mediated ROS in the cytoplasm, leading to RIPK1-dependent necroptosis, in L929 cells (80, 81). Moreover, lipid peroxides can induce necroptosis in erythroid cells lacking glutathione peroxidase 4 (GPX4) by acting upstream of the necrosome independent of TNFα treatment (82). GPX4 was found to be required for preventing RIPK3-dependent necroptosis in erythroid precursor cells by preventing lipidic ROS accumulation (82).

Pyroptosis, dependent on caspase, is a mode of inflammatory cell death in innate immune cells triggered by pathogen-associated molecular patterns (PAMPs) or damage-associated molecular patterns (DAMPs). Not only can PAMPs such as lipopolysaccharide (LPS) directly activate caspase-4 and -5 (in humans) and -11 (in mice) (83), inflammatory signals can be identified by intracellular sensor molecules such as NLR family pyrin domain containing 3 (NLRP3), resulting in caspase-1 activation through activation of the inflammasome. Finally, inflammasome arousal leads to the activation of the gasdermin (GSDM) family member protein gasdermin-D (GSDMD), which is responsible for pyroptosis through its pore-forming function on the plasma membrane (84). Cancer cells, including PDAC cells, also undergo pyroptosis. Macrophage stimulating 1 (MST1), a kinase that facilitates caspase-1-dependent pyroptosis by increasing the production of ROS, is usually downregulated in PDAC cells (85). GPX4 also plays an important role in protecting cells against GSDMD-mediated pyroptosis by negatively regulating lipid peroxidation as well as blocking GSDMD cleavage and activation (86).

#### 4.4.3 Ferroptosis Connects Redox and Cell Death

Ferroptosis is the type of RCD most inseparably connected to redox regulation and has emerged as a critical modality in different cancers. Ferroptosis is a form of iron- and ROS-dependent cell destruction driven by excessive lipid peroxidation (87). Unrestrained lipid peroxidation is the hallmark of ferroptosis. Both non-enzymatic and enzymatic catalysis can promote lipid peroxidation (88). The acyl-CoA synthetase long-chain family member 4 (ACSL4)–lysophosphatidylcholine acyltransferase 3 (LPCAT3)–arachidonate lipoxygenase (ALOX) axis plays an essential role in inducing ferroptosis by excessive lipid peroxidation to generate phospholipid hydroperoxide (PLOOH) from PUFAs in membrane phospholipids (89). Additionally, as an enzymatic-independent way, the increased intracellular iron reacts with H_2_O_2_, generating •OH and promoting excessive oxidation of PUFAs, which leads to further oxidation of lipids and produces toxic by-products. Promoting the degradation of the intrinsic iron storage protein ferritin or the cytosolic iron exporter solute carrier family 40 member 1 (SLC40A1, as known as ferroportin-1) *via* autophagy can induce and increase ferroptosis (90). Metastatic melanoma cells strive to limit lipid peroxidation by upregulating the expression of transferrin, which decreases intracellular iron levels and mitigates lipid oxidative stress (91), and by replacing PUFAs with monounsaturated fatty acids (MUFAs) to bind to membrane lipids, reducing the oxidation of PUFAs (63).

**Figure 4:**
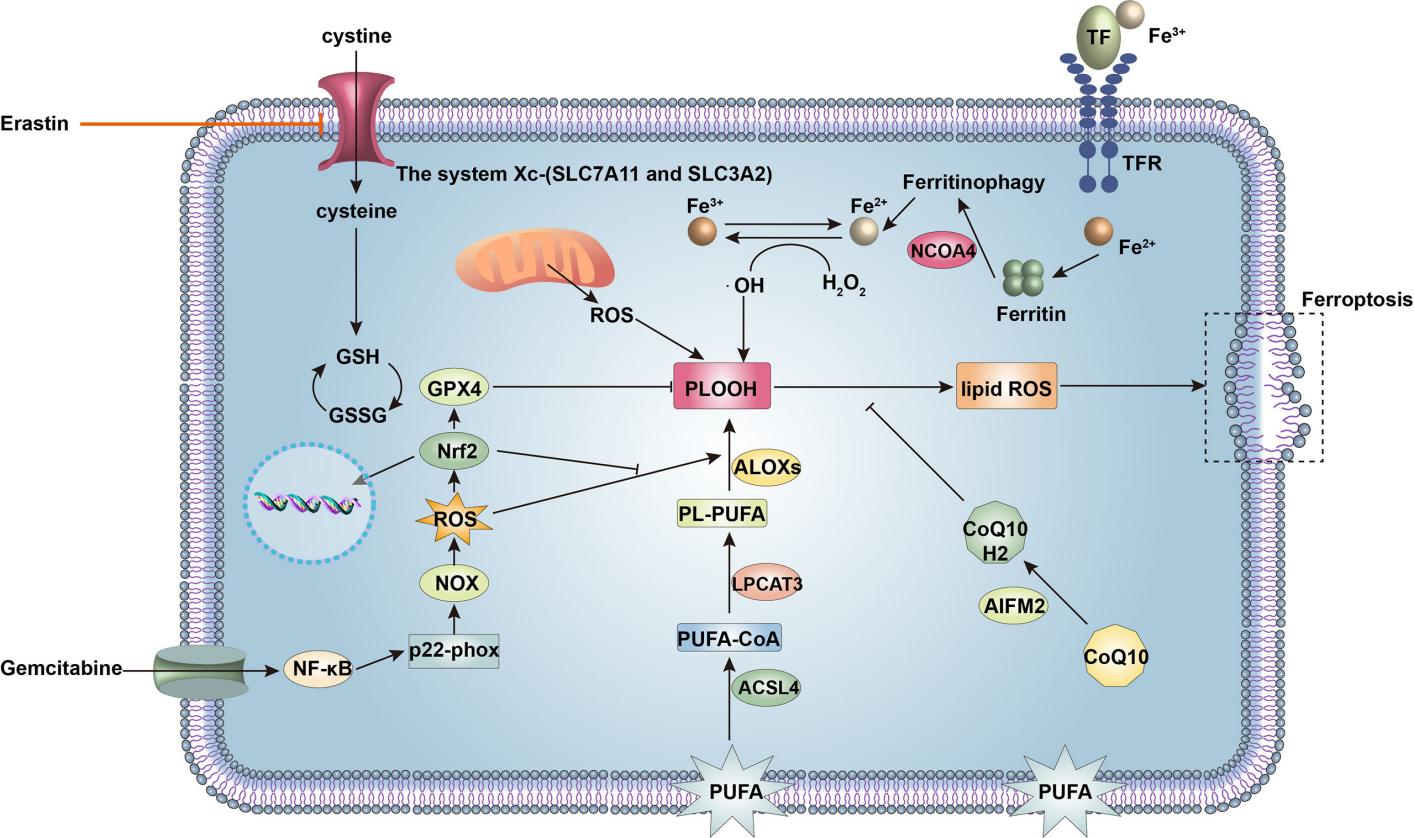
Signaling molecules and pathways regulating ferroptosis and gemcitabine resistance in cancer cells. Ferroptosis is a form of cell death driven by iron accumulation and lipid peroxidation. The acyl-CoA synthetase long-chain family member 4 (ACSL4)-lysophosphatidylcholine acyltransferase 3 (LPCAT3)-arachidonate lipoxygenases (ALOXs) pathway enzymatically mediates oxidation of polyunsaturated fatty acids (PUFAs) of the plasma membrane to phospholipid hydroperoxide (PLOOH), ultimately leading to ferroptosis. Nuclear receptor coactivator 4 (NCOA4) induces ferritinophagy to increase intracellular iron, promoting non-enzymatic lipid peroxidation and inducing ferroptosis. However, glutathione peroxidase 4 (GPX4) inhibits lipid peroxidation together with glutathione (GSH), which prevents ferroptosis. Intracellular levels of GSH are maintained by the system *X*_*c* ^−^_ (solute carrier family 7 member 11, SLC7A11 and solute carrier family 3 member 2, SLC3A2), which accepts extracellular cystine for GSH synthesis. In addition, apoptosis-inducing factor mitochondrial 2 (AIFM2) maintains a reduced state of coenzyme Q_10_ (CoQ10H2) to prevent lipid peroxidation and ferroptosis. Therefore, upregulation of SLC7A11, GSH, GPX4, and AIFM2 expression renders cancer cells resistant to ferroptosis. Gemcitabine can increase reactive oxygen species (ROS) production *via* nuclear factor kappa-B (NF-κB)-p22^-phox^-nicotinamide adenine dinucleotide phosphate oxidase (NOX) pathway in pancreatic ductal adenocarcinoma (PDAC) cells to induce ferroptosis. Activation of nuclear erythroid 2-related factor (Nrf2) decreases ROS and suppresses ferroptosis.

GPX4 is a glutathione peroxidase that serves to protect cells against excessive membrane lipid peroxidation by detoxifying and neutralizing lipid ROS (92). GPX4 requires GSH synthesized from the amino acid cysteine, which is generated by the reduction of cystine taken up by the cell. System $X_{c^{-}}$ , composed of the SLC7A11 and SLC3A2 proteins, is an amino acid antiporter that exchanges extracellular cystine and intracellular glutamate across the cell membrane (77). This system maintains the intracellular levels of GSH and GPX4 by maintaining the intracellular concentration of cysteine and promotes ferroptosis resistance. Inhibition of system $X_{c^{-}}$ results in consumption of cysteine and GSH and induces ferroptosis in PDAC cells (93). GSH acts as a cofactor for GPX4 to detoxify lipid peroxides during ferroptosis. Mutations in Kras occur in more than 95% of PDACs, and these tumors are characterized by high ROS levels. To eliminate ROS and prevent widespread cellular damage, PDAC cells upregulate GSH to evade ferroptosis. Additionally, apoptosis-inducing factor mitochondrial 2 (AIFM2), now appropriately renamed ferroptosis suppressor protein 1 (FSP1), plays a critical GSH-independent role in suppressing ferroptosis (94). Specifically, FSP1 acts to maintain a reduced state of coenzyme Q_10_ (CoQ), which traps lipid radicals at the plasma membrane and prevents lipid peroxidation. FSP1 expression correlates positively with resistance to ferroptosis in hundreds of different cancer cell lines (95) (**Figure 4**).

Tumor protein p53 (TP53) is a tumor suppressor gene expressing p53, which regulates DNA repair, the cell cycle, cell apoptosis, and senescence. TP53 can also regulate ferroptosis, as it was shown that p53 transcriptionally represses the expression of SLC7A11, thus reducing cystine uptake and promoting ferroptosis in lung cancer cells experiencing oxidative stress (96). Moreover, the P53^R273H^ and P53^R175H^ mutants were shown to inhibit SLC7A11 expression in mice by binding and segregating the transcription factor Nrf2 (97). However, the TP53^P47S^ mutant can endow cells with resistance to ferroptosis *in vitro*, and mice harboring the TP53^P47S^ mutation are susceptible to PDAC because of the decreased ability of the TP53^P47S^ mutant to inhibit SLC7A11 (98). Furthermore, abnormal expression of some ferroptosis-related TP53 target genes has been identified in cancer cells, including downregulation of the proferroptotic gene SAT1 (99) and upregulation of the antiferroptotic gene CDKN1A/p21 (100), which contributes to inhibiting ferroptosis in cancer cells and maintaining their survival. Therefore, the relationship between ferroptosis and TP53 expression in cancer cells shows that cancer cells can control ferroptosis by maintaining redox homeostasis at the DNA, mRNA, and protein levels.

Overall, although excessive ROS-induced RCD serves as a defense mechanism against the success of cancer, numerous mechanisms can be exploited by cancer cells to protect themselves from death and facilitate their initiation and maintenance as well as their therapeutic resistance. Maintaining homeostasis through redox regulation is one strategy by which cancer cells avoid RCD.

### 4.5 Autophagy Interacts With Redox Regulation in Cancer Cells

Autophagy is a catabolic phenomenon by which intracellular molecules and organelles are engulfed and encapsulated into double-membrane vesicles called autophagosomes, which interact with lysosomes for degradation of their components, thereby recycling intracellular components and maintaining cellular homeostasis (101). Autophagy not only happens at a basic level in cells but also can be triggered by diverse signaling pathways and cellular regulators and result in selective or nonselective degradation. Just as oxidative stress has a dual effect on tumor initiation and development, autophagy also has different effects on cancer based on the context, such as the stage of cancer development or the level of autophagy. Autophagy is believed to prevent cancer initiation by removing oncogenic molecules, toxic unfolded proteins, and dysfunctional organelles, but once the tumor forms, increased autophagic levels typically allow cancer cells to survive, grow, and migrate (102).

Autophagy is regulated by a rigorous and precise set of signaling pathways mainly involving autophagy-related genes (ATGs). The process of autophagy consists of five stages: initiation, phagophore nucleation, phagophore elongation, phagophore fusion, and content degradation. Autophagy is initiated upon the release of the ULK1 (ATG1) complex from mTOR inhibition. The ULK1 complex, consisting of ULK1, ULK2, FIP200, ATG101, and ATG13, leads to phagophore nucleation, which is subsequently induced by a class III PI3K complex that comprises VPS15, VPS34, ATG14, Beclin 1, UVRAG, and AMBRA1. ULK1 phosphorylates Beclin 1, which performs as a protein scaffold for the PI3K complex and accelerates the recruitment of other proteins in the phagophore. Two ubiquitin-like conjugation systems participate in phagophore elongation to form autophagosomes. The first conjugation system is the association of phosphatidylethanolamine (PE) with intracellular LC3-I to form LC3-II, the lipidated form of LC3; this conversion is promoted by ATG4B, ATG3, and ATG7 and results in the incorporation of LC3-II into the growing double membrane. The second system is mediated by ATG7 and ATG10 and results in the formation of the ATG5–ATG12 complex. Finally, the protein syntaxin 17 (STX17) induces the fusion of the autophagosome with the lysosome, leading to the formation of the autophagolysosome and degradation of the autophagosome contents (103).

Autophagy has a complicated relationship with redox regulation in cancer cells. Oxidative stress can induce autophagy in cancer cells. H_2_O_2_ can promote the accumulation of LC3-PE by inhibiting the delipidating activity of ATG4 in the cytoplasm and induce autophagy in cells during amino acid starvation (104). In MCF-7 breast cancer cells, oxidative stress activates the transcription factor forkhead box O3 (FOXO3), stimulating the transcription of LC3 and adenovirus E1B 19-kDa-interacting protein 3 (BNIP3), which are involved in autophagy (105). Moreover, oxidative stress can induce autophagy through AMP-activated protein kinase (AMPK). AMPK is sensitive to ROS and can be phosphorylated by the upstream kinase AMPK kinase (AMPKK) following H_2_O_2_ accumulation (106). Activated AMPK not only directly phosphorylates and activates ULK1 to induce autophagy, but also inhibits mTORC1 through phosphorylation of TSC2 and raptor during glucose deprivation, thereby reducing the inhibitory effect of mTORC1 on ULK1 (107).

From the perspective of the tumor-promoting effect of autophagy, autophagy can be considered a mechanism by which cancer cells resist oxidative stress. Cancer cells in hypoxic regions, characterized by high levels of ROS, have high autophagic flux to promote their survival, which may prevent an increase in ROS production (108). Hypoxia-inducible factor-1α (HIF-1α) is a major factor used by cells to adapt to hypoxia and oxidative stress. HIF-1α induces the transcription of the Bcl-2 and BNIP3 genes, whose expressed proteins compete with beclin-1 to combine with BCL2, thereby increasing cellular beclin-1 and promoting autophagy (109). Moreover, HIF-1α triggers selective mitophagy through the expression of BNIP3 to prolong cell survival during hypoxia (110). Accordingly, HIF-1α-induced autophagy and mitophagy reduce ROS levels in cancer cells during hypoxia to promote cell survival. Moreover, studies have shown that PDAC cell lines exhibit high autophagic flux and that downregulation of autophagy following ATG5 or ATG7 knockout decreases PDAC cell survival (111). This effect occurs partially because autophagy can maintain cellular redox homeostasis by preventing the accumulation of cytotoxic amounts of ROS, thereby allowing continuous tumor growth. Impairment of autophagy in these cells is associated with excessive oxidative stress, increased mitochondria impairment and decreased cell survival (112). Mitophagy, a subtype of selective autophagy, is a way to eliminate ROS and mitigate oxidative stress for cancer cells. In addition to the abovementioned BNIP3-induced mitophagy, membrane depolarization of dysfunctional mitochondria activates PTEN-induced putative kinase 1 (PINK1), which induces the activation of the E3 ligase parkin (PARK2) to ubiquitinate mitochondrial outer membrane protein substrates, providing a recognition signal for the autophagic machinery (113). This in turn leads to selective clearance of damaged mitochondria and decreases ROS production. Therefore, in cancer cells, ROS are eliminated through high autophagic flux to prevent the toxic effects of ROS, thereby promoting tumor progression.

Autophagy and Nrf2 are inextricably linked through p62. p62 is an adaptor protein that not only can induce autophagy, including that of itself and of other target proteins, but also can release Nrf2 from KEAP1 and exert its antioxidant effect by directly binding KEAP1 (114, 115). Meanwhile, the expression of p62 can also be regulated by Nrf2, indicating that there is a positive feedback mechanism in the p62–KEAP1–Nrf2 axis (116). As p62 is an autophagy substrate, inhibition of autophagy leads to accumulation of p62 and subsequent activation of Nrf2. Liver-specific deletion of autophagy gene ATG7 was shown to cause p62 accumulation, translocation to the nucleus of Nrf2, overexpression of Nrf2-target genes, and carcinogenesis, which was prevented by p62 deficiency (117). Moreover, deficiency of p62 and Nrf2 was found to greatly suppress the progression of oncogenic RAS-driven NSCLC in mouse models (27, 118). These lines of evidence show the oncogenic roles of p62 and Nrf2. Based on this context combined with the stage of cancer initiation, autophagy may play a role in suppressing tumorigenicity. Autophagy inhibits the occurrence of tumors by degrading p62 and then inhibiting Nrf2 activation. Therefore, as mentioned above, autophagy not only plays the role of scavenging ROS to protect cancer cells under the induction of oxidative stress, but also can inhibit tumorigenesis by degrading carcinogens.

### 4.6 Metabolic Reprogramming for Redox Homeostasis

Cancer cells are highly metabolically active and undergo metabolic reprogramming by which they can autonomously modify their metabolic efficiency and activity to satisfy the increased biosynthetic demand as well as mitigate oxidative stress, which is required for their proliferation and metastasis.

The discovery of the Warburg effect produced a torrent of cancer metabolism research and showed that cancer cells utilize glucose differently than normal cells. Cancer cells exhibit high uptake of glucose and preferential metabolism of glucose *via* glycolysis instead of mitochondrial OXPHOS, even in the presence of adequate oxygen; this preference is a common metabolic characteristic of cancer cells (119). How do cancer cells achieve this state of high glucose uptake and glycolysis? Dysregulated expression of genes encoding key glycolytic enzymes has been observed in cancer cells. The oncogene MYC has been shown to upregulate lactate dehydrogenase A (LDHA), an important glycolytic enzyme that is necessary to increase the glycolytic rate and tumorigenic potential of cancer cells (120). HIF1 induces an increase in glucose transporter 1 (GLUT1) activity, resulting in increased glucose intake (121). These mechanisms increase glycolysis through transcriptional upregulation of glucose transporters and glycolytic enzymes.

Why do cancer cells exhibit the Warburg effect, which uses more glucose but inefficiently produces ATP? This question has been studied in recent decades and urgently needs to be answered. From the perspective of cellular redox, the Warburg effect has been considered to favor cancer cells because a high level of glycolysis in cancer cells can decrease ROS generation and the induction of oxidative stress. Compared with untransformed cells, cancer cells have defects in mitochondrial metabolism (122). Glucose deprivation more readily induces oxidative stress in cancer cells, thereby increasing the susceptibility of cancer cells to glucose deprivation-induced cytotoxicity, which is detrimental to cancer cells (123). Although oxidative stress following glucose deprivation induces activation of Lyn kinase (Lyn) and JNK1 and increases the expression of c-Myc (124), cancer cells require increases in glucose metabolism to compensate for this defect (125). Cancer cells express high levels of pyruvate dehydrogenase kinases (PDKs) to inhibit PDH, which is responsible for catalyzing the conversion of pyruvate to acetyl-CoA, which flows into the tricarboxylic acid (TCA) cycle (126). By inhibiting PDH, detached cancer cells steer glucose away from the TCA cycle, thereby reducing the production of mitochondrial ROS, limiting the cytotoxic effects of oxidative stress, and inducing resistance to anoikis, a form of programmed cell death in which cells detach from the extracellular matrix (ECM). The inhibition of PDKs and activation of PDH can make some cancer cells sensitive to anoikis and decrease their metastatic efficiency due to ROS production (126). Moreover, upregulation of MnSOD sustains the Warburg effect through activation of the AMPK pathway to enhance the malignancy of tumor cells (127). Therefore, cancer cells can maintain redox homeostasis through the Warburg effect to acquire a survival advantage by restricting mitochondrial respiration and decreasing the generation of mitochondrial ROS.

On the other hand, cancer cells metabolize glucose through the PPP to support redox homeostasis. The PPP is the main source of NADPH, which can neutralize ROS and maintain intracellular levels of GSH (128). Glycolytic enzymes such as pyruvate kinase (PK) are involved in redirecting glycolytic flux through the PPP for detoxification of ROS. In cancer cells, ROS inhibit pyruvate kinase M2 (PKM2) to divert glucose flux into the PPP and thereby generate NADPH (129). Therefore, glucose metabolic reprogramming, including glycolysis and PPP activity, is involved in decreasing ROS levels in cancer cells.

Another metabolic pathway intimately related to redox homeostasis in cancer cells is glutaminolysis. Glutaminolysis is a metabolic process by which glutaminase (GLS) catalyzes the formation of ammonia and glutamate from glutamine. Then, glutamate is metabolized by glutamate dehydrogenase (GDH1) to alpha ketoglutarate (α-KG), a TCA cycle intermediate (130). Cancer cells exhibit high levels of glutamine uptake and glutaminolysis. The transcription factor c-Myc is exploited to increase glutamine input by increasing the expression of glutamine transporters including system N transporter 2 (SN2) and alanine-serine-cysteine transporter 2 (ASCT2) in cancer cells (131). In addition, c-Myc promotes glutaminolysis by upregulating the expression of GLS and GDH1 in cancer cells (132). Moreover, the glutaminolysis pathway is triggered by the oncoprotein K-Ras in PDAC (133).

Increased glutaminolysis is also an essential component of tumor metabolic reprogramming. Why are cancer cells so addicted to glutaminolysis? In addition to providing the glutaminolysis product α-KG to flow into the TCA cycle to meet the anabolic needs of cancer cells, glutaminolysis can also directly or indirectly participate in ROS detoxification and resistance to oxidative stress. On the one hand, glutaminolysis generates antioxidants that protect cancer cells against oxidative stress. The production of reduced GSH, composed of glutamate, cysteine, and glycine, therefore depends on glutaminolysis to provide glutamate and on the $X_{c^{-}}$ antiporter to import the precursor of cysteine, cystine. Increased glutaminolysis is beneficial to the survival of sorafenib-resistant HCC cells *via* the NADPH-dependent antioxidative defense (134). On the other hand, glutaminolysis produces the intermediate α-KG for the TCA cycle to support a process called glutamine anaplerosis. This process can also generate other antioxidant molecules. For instance, the process of converting malate to oxaloacetate (OAA) in the TCA cycle can simultaneously reduce NADP+ to NADPH (135). In addition to producing antioxidants, metabolites of glutaminolysis in the TCA cycle can counteract oxidative stress through enzymes or transcription factors. For instance, α-KG can bind to calcium/calmodulin-dependent protein kinase kinase 2 (CAMKK2) and stimulate the activity of AMPK, a substrate of CAMKK2, thereby inhibiting the mTOR pathway, balancing redox status and ultimately reactivating anoikis resistance signaling in LKB1-deficient lung cancer (136). Fumarate generated in the TCA cycle can interact with and increase the activity of GPX1 to use GSH to eliminate excessive ROS (137). Furthermore, fumarate can modify the cysteine residues on KEAP1 to activate the antioxidant response of Nrf2 (138). In general, glutaminolysis is necessary for TCA anaplerosis, maintenance of the bioenergetic capacity, and regulation of redox status in diverse kinds of cancer.

### 4.7 The Tumor Microenvironment Required for Tumor Promotion

The TME, composed of tumor cells, immune and inflammatory cells, fibroblasts, the ECM, tumor lymphatics, capillaries, and various cytokines and chemokines, is a complex integrated system. It takes part in many aspects of tumors and generates the tumor vasculature, which is closely implicated in metastatic progression. Synergistic interplay between cancer cells and cells within the TME, such as cancer-associated fibroblasts (CAFs) and immune cells, forms a tumor-promoting microenvironment. Redox regulation in cancer cells participates in this process, and the TME in turn maintains redox homeostasis cancer cells.

#### 4.7.1 ROS Affect CAFs in the TME

*Via* tumor–stroma coevolution, cancer cells induce oxidative stress in adjacent fibroblasts by secreting H_2_O_2_ to simulate a hypoxic environment (139). Consistent with this observation, when MCF7 cells were cocultured with CAFs, ROS production spreads laterally from the cancer cells to the CAFs under the action of H_2_O_2_ initially generated and secreted by the cancer cells (140). In addition, the cocultured CAFs displayed an increase in glucose uptake and a corresponding decrease in mitochondrial activity. Mitochondrial activity increased, glucose uptake decreased, and GLUT1 expression was downregulated in MCF7 cells cocultured with fibroblasts (140). Notably, this crosstalk between MCF7 cells and CAFs was abolished by the addition of CAT. Furthermore, caveolin-1 (Cav-1) was downregulated in fibroblasts cocultured with breast cancer cells (141). Cav-1 downregulation is associated with early tumor recurrence, metastasis, and poor clinical outcomes in human breast cancer patients (142). Cav-1 negatively regulates the uptake of exosomes; thus, loss of Cav-1 leads to increased uptake of exosomes into fibroblasts (143). Then, the increased exosomal influx reprograms fibroblasts to more protumorigenic CAFs by remodeling of the actin cytoskeleton, altering the gene expression and inducing an inflammatory phenotype in target cells, and inducing the release of cytokines and proangiogenic factors (144). This observation explains why a decrease in Cav-1 expression is associated with a high degree of tumor malignancy. Degradation of Cav-1 is induced by autophagy mediated by oxidative stress-induced activation of HIF1α and NFκB (145). Taken together, cancer cells redoxly interact with CAFs to alleviate oxidative stress and promote the formation of a tumor-supportive microenvironment.

Additionally, ROS derived from chronic oxidative stress caused by inactivation of junD have been shown to drive the differentiation of fibroblasts into highly migrating myofibroblasts through accumulation of the transcription factor HIF-1α and the chemokine CXCL12 (146). Myofibroblasts, which express α-smooth muscle actin (α-SMA), are associated with stimulation of tumor growth and invasion, enhanced metastatic spread, and shortened patient survival (146). Transforming growth factor β (TGF-β) and stromal cell-derived factor 1 (SDF-1) autocrine signaling loops stimulate each other to induce the transition from fibroblasts to myofibroblasts in a ROS-dependent manner. TGF-β autocrine signaling induces both Smad signaling and SDF-1-CXCR4 autocrine signaling pathway to promote myofibroblast differentiation. On the other hand, activation of SDF-1-CXCR4 signaling can also upregulate the expression of TGF-β (147). This evidence indicates that a high level of ROS in the TME promotes myofibroblast differentiation to form a tumor-promoting microenvironment and support tumor infiltration and metastasis.

#### 4.7.2 ROS Regulate Immune Cells in the Immunosuppressive TME

High levels of ROS in the TME contribute to an immunosuppressive TME by interacting with numerous immune cells, such as tumor-infiltrating lymphocytes (TILs), tumor-associated macrophages (TAMs), myeloid-derived suppressor cells (MDSCs), and regulatory T cells (Tregs).

TAMs, MDSCs, and Tregs act as immunosuppressive cells in the TME and can be regulated by ROS to enhance immunosuppressive and tumor-promoting abilities. TAMs are macrophages differentiated from monocytes that infiltrate into tumor tissues. High ROS levels in primary melanoma increase the secretion of TNF-α by TAMs by enhancing peroxisome proliferator-activated receptor γ (PPARγ) translocation, regulated by MAPK/ERK 1 to promote cancer cell invasion (148). The TME induces a phenotypic switch in macrophages from the proinflammatory M1 phenotype to the immunosuppressive M2 phenotype, which subsequently results in T-cell suppression through the production of ROS (149). MDSCs are immunosuppressive, heterogeneous myeloid cells. MDSCs adapt to high concentrations of ROS in the TME through activation of the transcription factors Nrf2 and HIF-1α to maintain their T-cell-inhibitory effect. In addition, ROS derived from MDSCs can suppress T-cell responses and cytotoxicity in the TME (150). H_2_O_2_ released by MDSCs decreases CD3ζ expression in T cells, thereby limiting the activation of T cells and reducing their expression of IFN-γ (151). Other studies have shown that colorectal cancer cell-recruited MDSCs inhibit T-cell activity and promote cancer cell growth through oxidative metabolism to generate ROS (152). Tregs also have an immunosuppressive function in the TME. Tregs require mitochondrial complex III, a main source of ROS, to maintain the expression and suppressive function of immunoregulatory genes. Mice that lack mitochondrial complex III specifically in Tregs exhibit loss of T-cell suppressive capacity (153). Although apoptosis occurs in Tregs because of their weak Nrf2-associated antioxidant system and high vulnerability to ROS in the TME, apoptotic Tregs release and convert large levels of ATP to adenosine and mediate immunosuppression *via* this adenosine. Therefore, ROS-mediated apoptosis of Tregs has been shown to sustain and amplify their suppressive capacity more efficiently. In addition, these apoptotic Tregs abrogated the tumoricidal effect of PD-L1 blockade in mouse tumor models (154).

Tumor-infiltrating lymphocytes (TILs) are immune cells that have migrated from the bloodstream into a tumor. Tumor-infiltrating cytotoxic T cells such as CD8 T cells are required mainly for the antitumor immune response. However, T-cell cytotoxicity is inhibited in the TME to support tumor development. Although appropriate concentrations of mitochondrial ROS are essential for the activation and antitumor activity of T cells, high concentrations of ROS contribute to hyporesponsiveness of and functional damage to T cells. CD8 TILs in clear cell renal cell carcinoma (ccRCC) have small and fragmented mitochondria that generate large amounts of ROS. Elevated mitochondrial ROS levels impair CD8 T-cell function, although this effect can be mitigated by using mitochondrial ROS scavengers (155). Furthermore, tumor-infiltrating CD8 T cells with decreased mitochondrial function and mass exhibit loss of antitumor immune responses and responses to PD-1 blockade, although these losses can be reversed by overexpression of PGC1α, the major factor in mitochondrial biosynthesis (156). Therefore, the mitochondrial dysfunction in T cells in the TME and the consequent excessive generation of ROS lead to suppression of the tumor immune response.

Taken together, these findings suggest that the increased ROS level within the TME regulates various immune cells in order to suppress tumor immunity and facilitate tumor progression. Based on this effect of ROS on tumor immunity, we speculate that ROS may mechanistically mediate resistance to tumor immunotherapy and that targeting ROS may improve the efficacy of immunotherapy. A study in a breast cancer model showed that elimination of ROS in the TME by using advanced nanomaterials increased the infiltration of T cells and elicited antitumor immunity, resulting in highly potent antitumor effects and improving the efficacy of tumor immunotherapy (157). However, other research has proposed that in mouse models of programmed death-1 (PD-1) blockade therapy, tumor-reactive cytotoxic T lymphocytes (CTLs) harbor more ROS. Moreover, increased ROS production by ROS inducers or indirectly by mitochondrial uncouplers synergizes with the antitumor activity of PD-1 blockade by the expansion of intratumoral effector/memory CTLs (158). This effect may be related to the stage at which ROS act on T cells in the TME and the source of the ROS. The high level of ROS in the TME hinders the activation of T cells, but when T cells mediate tumor immunity, they generate ROS to activate tumoricidal pathways. Maintaining the integrity of mitochondrial function in T cells is important. Simultaneously, we cannot ignore the observation that the effectiveness of immunotherapies in exerting their antitumor effects depends on a fully functional immune system. The tumor immune function of T cells is regulated by various factors, and ROS may directly or indirectly affect the immune system either positively or negatively.

## 5 The Redox System and Therapy and Resistance in Cancer

### 5.1 Therapies Targeting the Redox System

In the past, through studies based on the carcinogenic effects of ROS, dietary supplementation with antioxidants was believed to be able to prevent or treat cancer by reducing ROS levels. The effectiveness of antioxidants in cancer treatment has been proven in numerous animal models and *in vitro* studies. For example, *in vitro*, overexpression of MnSOD or CuZnSOD significantly decreased breast cancer cell growth (159). The combination of N-acetylcysteine (NAC) and vitamin C was found to prevent the onset of cancer in a model of MYC-dependent human B lymphoma (160). However, the results of many clinical trials launched to confirm whether dietary supplementation with antioxidants can reduce cancer incidence or inhibit tumor progression were disappointing. Large-scale clinical trials indicating that long-term vitamin E supplementation does not prevent cancer have been published (161). The SELECT study even demonstrated that dietary supplementation with vitamin E significantly increased the risk of prostate cancer among healthy men (162). Moreover, many studies on antioxidant dietary supplementation have demonstrated insufficient evidence that antioxidants can prevent cancer or suppress cancer progression (163). Thus, the hope of using antioxidant dietary supplementation to prevent cancer has waned. However, the use of some antioxidants in combination with traditional radio-chemotherapies appears promising in the treatment of cancer. A phase I clinical trial of ascorbic acid with gemcitabine in the control of metastatic and node-positive pancreatic cancer showed well-tolerated and some preliminary efficacy (164). Results from a phase I clinical trial of pharmacological ascorbate combined with radiation and temozolomide for newly diagnosed glioblastoma showed that the combination is safe and warrants further investigation (165). Antioxidants combined with radiotherapy may also reduce the incidence of side effects. Results of a phase IIb randomized double-blind trial of GC4419 (a superoxide dismutase mimetic) versus placebo showed that GC4419 significantly reduced severe oral mucositis induced by concurrent radiotherapy and cisplatin in head and neck cancer (166). Certainly, these clinical studies require further investigation to confirm the therapeutic effect of antioxidants combined with chemoradiotherapy.

Considering that cancer cells upregulate antioxidant production to eliminate excess ROS and maintain redox homeostasis, prooxidant therapy has been confirmed to treat tumors at the redox level, which means that this therapy works by increasing ROS levels and exacerbating oxidative stress in cancer cells to promote their death and inhibit tumor progression. Widely used chemotherapeutic agents, including procarbazine, paclitaxel, daunorubicin, doxorubicin, alkylating agents, cisplatin, carboplatin, topotecan, and irinotecan, can increase ROS in cancer cells and kill these cells by exacerbating oxidative stress (167). Some selective drugs that inhibit mitochondrial SDH, such as α-Tocopheryl succinate (α-TOS), have also been widely studied, which can induce cancer cell apoptosis by targeting SDH to generate ROS (168). On the other hand, in addition to production of excessive ROS, targeted suppression of the antioxidant system in cancer cells is another element of prooxidant therapy. Some small-molecule drugs with prooxidant effects have also been widely studied. Erastin induces ferroptosis by selectively inhibiting system $X_{c^{-}}$ and decreasing GSH synthesis. In one study, folate-targeting exosomes were used to deliver erastin to triple-negative breast cancer cells. This folate-vectorized exosome-encapsulated erastin selectively targeted MDA-MB-231 cells and promoted ferroptosis through intracellular GSH depletion and ROS overproduction, inhibiting the proliferation and migration of these cells (169). Brusatol is an inhibitor of the Nrf2 pathway. A study showed that brusatol caused quick and transient exhaustion of the Nrf2-related protein in Hepa-1c1c7 mouse hepatic carcinoma cells and freshly isolated primary human hepatocytes, thereby sensitizing these cells to the cytotoxic effects of other chemotherapeutic drugs (170). Imexon is a prooxidant molecule that exhausts GSH, blocks GPX1 activity, and increases ROS levels; its activity against non-Hodgkin lymphoma was evaluated in a phase II trial (171).

Taken together, these findings indicate that regardless of the method or drug, it is important to destroy the redox homeostasis that cancer cells have painstakingly established. Specific and carefully adjusted interventions provide the opportunity to disrupt redox homeostasis in cancer cells. With the continuous increases in the understanding of cancers and the continuous advances in research methods and technologies, novel redox-based therapeutic approaches can improve the therapeutic effect on tumors and improve patient prognosis.

### 5.2 Therapeutic Resistance in Cancer Cells

Unfortunately, cancer cells acquire resistance to chemotherapy, radiotherapy, or immunotherapy through redox regulation. Cancer cells upregulate antioxidant enzymes to develop resistance to antitumor therapies. The expression of GSH has been shown to be upregulated in NSCLC, leading to resistance to cisplatin therapy (172). Upregulation of Nrf2 is also a major cause of drug resistance in cancer cells, as Nrf2 is activated to increase antioxidant levels to detoxify ROS in cancer cells. Moreover, FOXO1 is implicated in drug resistance in cancer cells. FOXO1 activation *via* SIRT1-mediated deacetylation was observed to trigger overexpression of multidrug resistance protein 2 (MRP2) in tamoxifen-resistant MCF-7 breast cancer cells (173). Our group has explored the association between gemcitabine resistance and ferroptosis in PDAC; thus, here, we focus on discussing gemcitabine resistance, ferroptosis, and redox regulation in PDAC.

During the past two decades, gemcitabine has been the gold standard for systemic treatment of PDAC (174). The traditional anticancer mechanism of gemcitabine is to convert into gemcitabine diphosphate (dFdCDP) and gemcitabine triphosphate (dFdCTP) to block DNA extension and synthesis (175), but from a redox perspective, gemcitabine can generate ROS like other chemotherapeutic drugs. One of the mechanisms by which gemcitabine generates ROS is to induce NOX-derived ROS generation through an increase in the expression of p22^-phox^ *via* NF-κB activation (176). Thus, gemcitabine may also exert antitumor effects through the cytotoxic effect of excessive ROS, and this effect may also be related to ferroptosis (as discussed below).

However, the emergence of resistance to gemcitabine within weeks of treatment initiation has become a major obstacle in the treatment of PDAC with gemcitabine (177). On the one hand, this acquired resistance is related to the abundant fibrotic stroma of PDAC. The large amount of connective tissue surrounding the cancer cells may account for up to 90% of the total tumor volume and has been considered to form a physical obstacle to gemcitabine delivery (178). Among the various types of cell in the PDAC microenvironment, CAFs are the key fibrosis-generating cells. CAFs of PDAC are main secretory cells for soluble and insoluble ingredients that create the specific stroma that facilitates resistance to gemcitabine *via* physical barriers (177). Activated gemcitabine is entrapped within CAFs in the extracellular stroma, complicating its accessibility to cancer cells (179). On the other hand, PDAC cells possess intrinsic resistance to gemcitabine through the regulation of various molecular pathways. As a feedback mechanism, increased levels of gemcitabine-induced ROS activate Nrf2, which then triggers the transcription of cytoprotective antioxidant genes, especially genes encoding enzymes that catalyze GSH generation to eliminate the increased ROS (176). Furthermore, bioinformatic analysis showed that system $X_{c^{-}}$ (SLC3A2 and SLC7A11) and GPX4, the major negative regulators of ferroptosis, were upregulated in gemcitabine-resistant pancreatic cancer cells (180). Thus, gemcitabine-mediated ROS can be inferred to further induce ferroptosis in cancer cells, implying that we can enhance the efficacy of gemcitabine or reverse gemcitabine resistance by inducing ferroptosis through targeting Nrf2, SLC3A2, SLC7A11, and GPX4 (**Figure 4**). Knockdown of Nrf2 with Nrf2 siRNA showed that different PDAC cell lines were more sensitive to gemcitabine (176). Brusatol, an inhibitor of Nrf2, has been demonstrated to abrogate gemcitabine-induced Nrf2 activation, increase ROS accumulation, and potentiate gemcitabine-induced growth inhibition and cytotoxicity in pancreatic cancer cells (181). Another study demonstrated that plasma-treated water sensitizes pancreatic cancer cells to ferroptosis through targeted inhibition of Nrf2 and GPX4 (182). Therefore, it is worth further verifying whether combined treatment with Nrf2 inhibitors and ferroptosis inducers can reverse gemcitabine resistance and be a strategy for treating gemcitabine-resistant cells.

## 6 Conclusion: The Importance of Homeostasis and Context

From the origins of free radical theory of cancer to today, the research on redox metabolism of cancer cells has continued to progress and innovate. Initially, it was believed that ROS was carcinogenic and SOD played an important tumor suppressor role, and that scavenging ROS through SOD would inhibit cancer development. To confirm these ideas, the researchers did find decreased SOD expression in different cancers (183, 184), and found that SOD overexpression would inhibit cancer progression (185). However, with the extensive research and technological advancement, more and more studies have found that the expression of SOD in cancer is elevated, and this is beneficial to the development of cancer (186). Certainly, the development of these theories should be placed in the context of the stage of cancer development. Antioxidants inhibit the transformation of cells to a malignant state in the initiation and promotion phase of cancer development; however, once fully transformed, the cancer cells enter the progression phase, and during the invasion and metastasis phase of cancer, expression of antioxidants protects against the harsh microenvironmental conditions and is necessary to support the fully malignant phenotype. The switch occurs once the cancer cells overcome the stresses of the transformation process to survive and enter the phase of metastasis and rapid progression. To this day, research on redox metabolism in cancer cells continues, and for the evolution of this thinking, we emphasize the importance of redox homeostasis for cancer cells and the importance of disrupting this homeostasis for cancer treatment.

Redox homeostasis is critical to cancer cells. Throughout tumor progression, cancer cells must withstand oxidative stress during initiation, proliferation, matrix detachment, circulation, remote colonization, and treatment. Cancer cells develop various adaptive strategies to alleviate oxidative stress damage and limit ROS levels to a dynamic range that allows survival while promoting resistance to cell death. During initiation and progression, cancer cells usually contain a large amount of ROS and must thus strengthen their antioxidant defense, which usually requires overexpression of antioxidant genes regulated by Nrf2, as well as activation of other pathways that support increased production of antioxidants such as NADPH and GSH. During metastasis, cancer cells cooperate with CAFs and TAMs in the TME at the redox level to induce ROS-stimulated migration and further increase the production of antioxidants to reduce ROS-induced death. In the process of enduring oxidative stress, cancer cells also reprogram glucose metabolism to glycolysis and the PPP, reducing ROS generation and increasing NADPH production. The same is true for resistance to antitumor therapy; cancer cells overexpress various antioxidants to enhance the ROS detoxification ability of the antioxidant system. Therefore, cancer cells tend to maintain redox homeostasis in multiple stages. The antioxidant system in cancer cells maintains ROS at a level that is beneficial for the development of cancer cells.

Importantly, redox regulation in cancer cells is based on context. ROS do not indiscriminately exert carcinogenic effects, and Nrf2 does not indiscriminately exert tumor-suppressive effects. Every mechanism and molecule that we have discussed herein acts as a double-edged sword and plays opposite roles based on the context. During tumor initiation, H_2_O_2_ is an important protumorigenic signaling molecule. However, damaging ROS such as $O_{2^{-}}$ , -OH, and lipid hydroperoxide (LOOH) can be overproduced during tumor progression and promote the death of cancer cells. In addition, in preneoplastic cells, Nrf2 activation reduces inflammation and oxidative stress and reduces ROS-induced damage to DNA, thereby inhibiting cancer initiation. However, in the advanced stage of tumors, overexpression of Nrf2 reduces the level of ROS in cancer cells to protect cells against chemotherapeutic agents. Therefore, the importance of context should be considered in research. Different cancer types, different stages of tumor development, and different ROS concentrations, types, and sources lead to different results.

Finally, we must realize that although we have an understanding of cancer at the redox level, cancers are complex diseases involving multiple factors and multiple pathways in organisms. Thus, much remains to be done before problems can be completely solved at the etiology or treatment level. ROS and antioxidants may also play tumor-related roles in immune regulation. The traditional single increase in antioxidants such as dietary vitamin E may promote tumor metastasis through other mechanisms. Antioxidants may interfere with the ability of cells to sense oxidative stress. In addition, some molecular drugs targeting redox signaling pathways may also pass through or be affected by other pathways and fail to exert antitumor effects. Moreover, excessive ROS can cause severe damage to normal cells. Through continuous research and improved understanding of cancers, their mysteries will be revealed, and cancer treatment strategies will be further improved.

## Author Contributions

WW provided direction and guidance throughout the preparation of this manuscript. FX and QH wrote and edited the manuscript. YQ, JX, BZ, and XY reviewed and made significant revisions to the manuscript. All authors contributed to the article and approved the submitted version.

## Funding

This study was jointly supported by the National Natural Science Foundation of China (No. 82173178), the National Natural Science Foundation of China (U21A20374), Shanghai Municipal Science and Technology Major Project (21JC1401500), Scientific Innovation Project of Shanghai Education Committee (2019-01-07-00-07-E00057), Clinical Research Plan of Shanghai Hospital Development Center (SHDC2020CR1006A), and Xuhui District Artificial Intelligence Medical Hospital Cooperation Project (2021-011).

## Conflict of Interest

The authors declare that the research was conducted in the absence of any commercial or financial relationships that could be construed as a potential conflict of interest.

## Publisher’s Note

All claims expressed in this article are solely those of the authors and do not necessarily represent those of their affiliated organizations, or those of the publisher, the editors and the reviewers. Any product that may be evaluated in this article, or claim that may be made by its manufacturer, is not guaranteed or endorsed by the publisher.

## Glossary

**Table:**

ACSL4	acyl-CoA synthetase long-chain family member 4
AIFM2	apoptosis-inducing factor mitochondrial 2
Akt	protein kinase B
ALOX	arachidonate lipoxygenase
AMPK	adenosine 5‘-monophosphate (AMP)-activated protein kinase
APAF1	apoptotic peptidase activating factor 1
AP-1	activator protein 1
ARE	antioxidant response element
ASCT2	alanine-serine-cysteine transporter 2
ASK1	apoptosis signal-regulating kinase 1
ATGs	autophagy-related genes
ATP	adenosine triphosphate
BNIP3	adenovirus E1B 19-kDa-interacting protein 3
CAFs	cancer-associated fibroblasts
Cav-1	caveolin-1
CAMKK2	calcium/calmodulin-dependent protein kinase kinase 2
CAT	catalase
ccRCC	clear cell renal cell carcinoma
CoQ	coenzyme Q_10_
COX	cyclooxygenases
CTLs	cytotoxic T lymphocytes
DAMPs	damage-associated molecular patterns
DAO	diamine oxidase
DUOX1	dual oxidase 1
ECM	extracellular matrix
EGFR	epithelial growth factor receptor
ERK1/2	extracellular regulated protein kinases 1/2
EMT	epithelial–mesenchymal transition
ER	endoplasmic reticulum
FAD	flavin adenine dinucleotide
FADD	FAS-associated protein with death domain
FoxO3	forkhead box O3
FSP1	ferroptosis suppressor protein 1
GDH	glutamate dehydrogenase
GLUT1	glucose transporter 1
GLS	glutaminase
G6PD	glucose-6-phosphate dehydrogenase
GSH	glutathione
GPX	glutathione peroxidase
GSDM	gasdermin
GST	glutathione S-transferase
HIF-1α	hypoxia-inducible factor-1α
HO	heme oxygenase
H_2_O_2_	hydrogen peroxide
HOCl	hypochlorous acid
KEAP1	kelch-like ECH-associated protein 1
HCC	hepatocellular carcinoma
JNK	c-Jun N-terminal kinase
LC3	microtubule-associated protein light chain 3
LDHA	lactate dehydrogenase A
LKB1	liver kinase B1
LOX	lipoxygenases
LPCAT3	lysophosphatidylcholine acyltransferase 3
LPS	lipopolysaccharide
MAPK	mitogen-activated protein kinase
MCT1	monocarboxylate transporter 1
MDSCs	myeloid-derived suppressor cells
MLKL	mixed-lineage kinase domain-like pseudokinase
MMP-7	matrix metallopeptidase 7
Mn-SOD	manganese superoxide dismutase
MOMP	mitochondrial outer membrane permeabilization
MRP2	multidrug resistance protein 2
MST1	macrophage stimulating 1
mTOR	mammalian target of rapamycin
MUFAs	monounsaturated fatty acids
NAC	N-acetylcysteine
NADH	nicotinamide adenine dinucleotide
NADPH	nicotinamide adenine dinucleotide phosphate
NF-κB	nuclear factor kappa-B
NLRP3	NLR family pyrin domain containing 3
Nrf2	nuclear erythroid 2-related factor
NOX	NADPH oxidase
NSCLC	non-small cell lung cancer
OAA	oxaloacetate
OXPHOS	oxidative phosphorylation
O_2_	molecular oxygen
$O_{2}^{-}$	superoxide anion
^1^O_2_	singlet oxygen
•OH	hydroxyl radical
PAMPs	pathogen-associated molecular patterns
PDAC	pancreatic ductal adenocarcinoma
PDH	pyruvate dehydrogenase
PDK	pyruvate dehydrogenase kinase
PD-1	programmed death-1
PD-L1	programmed death ligand 1
PE	phosphatidylethanolamine
PGC-1α	peroxisome proliferator-activated receptor-γ coactivator-1α
PI3K	phosphatidylinositol 3-kinase
PINK1	PTEN -induced putative kinase 1
PK	pyruvate kinase
PKC	protein kinase C
PKM2	pyruvate kinase M2
PLOOH	phospholipid hydroperoxide
PON	paraoxonase
PPARδ	peroxisome proliferator-activated receptor δ
PPP	pentose phosphate pathway
PUFAs	polyunsaturated fatty acids
RCD	regulated cell death
RIPK1	receptor interacting serine/threonine kinase 1
ROS	reactive oxygen species
SDF-1	stromal cell-derived factor 1
SLC40A1	solute carrier family 40 member 1
SIRT1	sirtuin1
SN2	system N transporter 2
TAMs	tumor-associated macrophages
TCA	tricarboxylic acid
TGF-β	transforming growth factor β
TIGAR	TP53 induced glycolysis regulatory phosphatase
TILs	tumor-infiltrating lymphocytes
TME	tumor microenvironment
TNF	tumor necrosis factor
TNFR1	tumor necrosis factor receptor 1
TRAIL-R1/2	tumor necrosis factor-related apoptosis inducing ligand receptor 1/2
Tregs	regulatory T cells
Trx	thioredoxin
ULK1	unc-51 like autophagy activating kinase 1
VEGFC	vascular endothelial growth factor C
6PG	6-phosphogluconate dehydrogenase
8-oxodG	8-oxo-7-hydro-2′-deoxyguanosine
8-OHdG	8-hydroxy-2′-deoxyguanosine
